# Energy Management of Smart Home with Home Appliances, Energy Storage System and Electric Vehicle: A Hierarchical Deep Reinforcement Learning Approach

**DOI:** 10.3390/s20072157

**Published:** 2020-04-10

**Authors:** Sangyoon Lee, Dae-Hyun Choi

**Affiliations:** School of Electrical and Electronics Engineering, Chung-Ang University, 84 Heukseok-ro, Dongjak-gu, Seoul 156-756, Korea; sangyoon1207@naver.com

**Keywords:** home energy management, deep reinforcement learning, smart home appliance, energy storage system, electric vehicle

## Abstract

This paper presents a hierarchical deep reinforcement learning (DRL) method for the scheduling of energy consumptions of smart home appliances and distributed energy resources (DERs) including an energy storage system (ESS) and an electric vehicle (EV). Compared to Q-learning algorithms based on a discrete action space, the novelty of the proposed approach is that the energy consumptions of home appliances and DERs are scheduled in a continuous action space using an actor–critic-based DRL method. To this end, a two-level DRL framework is proposed where home appliances are scheduled at the first level according to the consumer’s preferred appliance scheduling and comfort level, while the charging and discharging schedules of ESS and EV are calculated at the second level using the optimal solution from the first level along with the consumer environmental characteristics. A simulation study is performed in a single home with an air conditioner, a washing machine, a rooftop solar photovoltaic system, an ESS, and an EV under a time-of-use pricing. Numerical examples under different weather conditions, weekday/weekend, and driving patterns of the EV confirm the effectiveness of the proposed approach in terms of total cost of electricity, state of energy of the ESS and EV, and consumer preference.

## 1. Introduction

Approximately 30 percent of the United States’ total energy consumption comes from the residential sector, and the amount of the residential energy consumption is expected to grow owing to increased use of home appliances (e.g., air conditioners (ACs) and washing machines (WMs)) and modern electronic devices [1]. Thus, an efficient and economical methodology for residential energy management is required to reduce the electricity bill of consumers and keep the efficiency of their appliances. Furthermore, as distributed energy resources (DERs) (e.g., a rooftop solar photovoltaic (PV), a residential energy storage system (ESS), and an electric vehicle (EV)) become integrated into an individual residential house via an advanced metering infrastructure with smart meters for reliable residential grid operations, the complexity of the residential energy management increases. The aforementioned challenges require more intelligent systems, i.e., home energy management systems (HEMSs), through which an electric utility or a third party provides consumers with an efficient and economical control of home appliances.

A primary goal of HEMS is to reduce the electricity bill of consumers while satisfying their comforts and preferences. To achieve this goal, HEMSs perform the following two functions: (1) real-time monitoring of the energy usage of consumers using smart meters; (2) scheduling of the optimal energy consumption of home appliances. To implement this second function, an HEMS algorithm is generally formulated as a model-based optimization problem. Recently, numerous studies have been published on the development of HEMS optimization algorithms [2,3,4,5,6,7,8,9,10,11,12]. These studies address the scheduling of the energy consumption for home appliances and DERs, while maintaining the consumer’s comfort level using mixed-integer nonlinear programming (MINLP) [2], the load scheduling using mixed-integer linear programming (MILP) for single and multiple households [3,4], robust optimization for scheduling of home appliances to resolve the uncertainty of consumer behavior [5], and distributed HEMS architectures consisting of local and global HEMSs [6]. A quickly distributed HEMS algorithm was developed for a large number of households using the MINLP approach with a nonconvex relaxation [7]. Based on an emerging technology for ESSs and EVs, a home energy consumption model under the control of the ESS was presented in [8]. A model predictive control-based HEMS algorithm was proposed using the prediction of the EV state [9]. An HEMS optimization model considering both ESS and EV was formulated for a single household [10,11] based on their bi-directional operation and multiple households with a renewable energy facility [12]. In addition, many studies proposed the methods to evaluate and preserve the consumer comfort during the HEMS process. In [13], a quality of experience (QoE)-aware HEMS was developed where the QoE-aware cost saving appliance scheduling and the QoE-aware renewable source power allocation are conducted to schedule the operation of the controllable loads based on the consumer preferences and the available renewable energy sources. A new demand management scheme based on the operational comfort level (OCL) of consumer was proposed to minimize the peak-to-average ratio while maximizing the OCL of consumers [14]. A score-based HEMS method was presented to maintain the total household power consumption below a certain limit by scheduling various household loads based on the consumer comfort level setting [15]. A QoE-aware HEMS algorithm was presented to reduce the peak load and electricity cost while satisfying the consumer comfort and QoE with a fixed threshold [16]. More recently, an advanced QoE-aware HEMS method considering renewable energy sources and EVs was developed for adaptively varying the QoE threshold [17]. Compared to the method with the fixed OoE threshold in [16], a fuzzy logic controller was designed to dynamically adjust the QoE threshold for optimizing the consumer QoE in [17]. A recent work on HEMS was summarized in [18].

However, the aforementioned optimization-based HEMS methods were executed according to deterministic equations to illustrate the operation characteristics of home appliances and DERs (e.g., consecutive operation time intervals of WMs and state of energy (SOE) dynamics of ESS) as well as consumer’s comfort level (e.g., preferred indoor temperature using indoor temperature dynamics). Consequently, the model-based HEMS optimization approach suffers from two limitations. First, the characteristics for the operation of appliances/DERs and consumer preference are expressed through approximated unrealistic equations with fixed parameters, thereby leading to an inaccurate energy consumption schedule. Second, an optimization method including a large number of decision variables could significantly increase the computation complexity and could not scale well with a greater number of houses. Furthermore, the solution resulting from model-based optimization may not always be guaranteed and often diverges owing to a smaller feasible region with a large number of operational constraints for the HEMS optimization problem. To address the aforementioned limitations, we propose a data-driven approach that leverages model-free reinforcement learning (RL) to calculate the optimal schedule of home energy consumption.

Recently, data-driven approaches based on various machine learning (ML) methods have gained popularity owing to their more efficient residential energy management. In [19,20], methods to predict the generation output of a PV system accurately during the day for efficient energy management of buildings were presented. These methods employed an artificial neural network (ANN) and a deep neural network (DNN), respectively. DNN methods were also used in [21,22,23] for load forecasting to minimize the energy usage of buildings and households. More recently, RL has received attention as a promising ML method for the energy management of buildings and homes. A pioneering study on RL-based energy management is Google DeepMind, which was developed using RL and proved to decrease the electricity bill by cooling the data center by approximately 40%. Another RL-based method, referred to as Q-learning, was applied to HEMS problems. It was integrated with the ANN module for estimating the consumer’s comfort level, maintaining the energy efficiency of household appliances [24,25] and predicting the pricing in real time [26]. A new demand response strategy for HEMS was proposed through the combination of Q-learning and fuzzy reasoning, which reduces the number of state-action pairs and fuzzy logic for reward functions [27]. Furthermore, methods based on deep reinforcement learning (DRL) such as Deep Q-Network, and policy gradient, were applied for energy management of building [28,29] based on both discrete and continuous action spaces. A holistic DRL method for the energy management of commercial buildings was presented in [30] where Heating, Ventilation, and Air conditioning (HVAC) system, lighting, blind, and window systems are controlled to achieve energy savings within the buildings’ occupants comfort in terms of thermal, air quality, and illumination conditions. To resolve the limit of model-free DRL methods such as low sample efficiency, a model-based RL method was developed for building HVAC control that trains the system dynamics using neural networks [31]. Based on the trained system dynamics, the operation of the HVAC system was managed by model predictive control to minimize both the energy cost and the indoor temperature constraints violation. The DRL approach was also applied to data centers with servers, which aim to minimize the energy used for moving air and on-demand cooling in the data centers through the control of the temperature and relative humidity of air supplied to the server [32].

Recent studies addressed on energy management systems for buildings and households using an RL-based method. However, to the best of the authors’ knowledge, no study has presented an DRL-based algorithm that considers the continuous operations of heterogeneous home appliances and DERs according to the consumer’s comfort and preferences. In prior studies [24,25,26], the operations of home appliances and DERs were scheduled using a simple Q-learning method based on an unrealistic discrete action space. Furthermore, prior studies [28,29] focused on the scheduling of the energy consumption of buildings without considering the operation characteristics of home appliances in detail.

**Figure 1:**
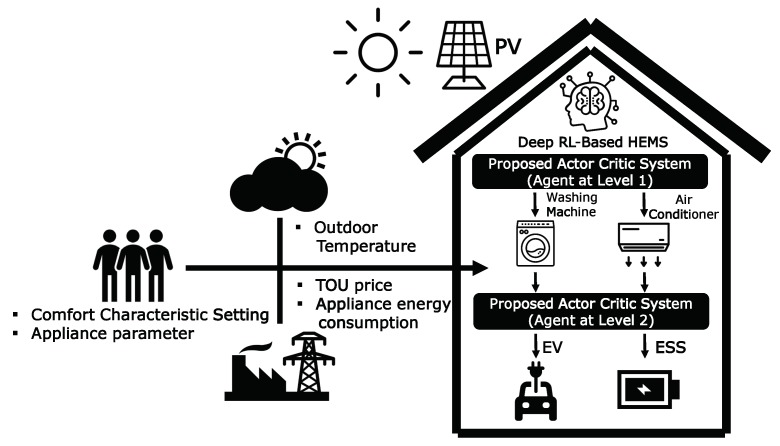
Conceptual architecture of the proposed deep reinforcement learning (DRL)-based home energy management system (HEMS) algorithm.

In this study, we propose a two-level DRL framework that employs an actor–critic method where the controllable home appliances (WM and AC) are scheduled at the first level according to the consumer’s preferred appliance scheduling and comfort level. The ESS and EV are scheduled at the second level to cover the aggregated WM and AC loads that are calculated at the first level along with the fixed load of the uncontrollable appliances. The proposed two-level scheme is motivated by the interdependent operation between the home appliances at the first level and the ESS/EV at the second level. If the proposed algorithm is executed in a single-level framework, the optimal policy for charging and discharging actions of the ESS and EV is independently determined without considering the energy consumption schedule of aggregated home appliances, thereby degrading the performance of the algorithm. Figure 1 presents the conceptual system model for the proposed two-level DRL-based HEMS that employs an actor–critic method, along with the data classification associated with the utility company, weather station, and consumer. The main contributions of this study are summarized as follows:

- We present a two-level distributed DRL model for optimal energy management of a smart home consisting of a first level for WM and AC, and a second level for ESS and EV. In such a model, the energy consumption scheduling at the second level is based on the aggregated energy consumption scheduled at the first level to determine the better policy of charging and discharging actions for the ESS and EV.
- Compared to the existing method using Q-learning in a discrete action space, we propose a hierarchical DRL in a continuous action space with the following two scheduling steps: (i) the controllable appliances including WM and AC are scheduled at the first level according to the consumer’s preferred appliance scheduling and comfort level; (ii) ESS and EV are scheduled at the second level, thereby resulting in optimal cost of electricity for a household.

The simulation results confirmed that the proposed HEMS algorithm can successfully schedule the energy consumptions of multiple home appliances and DERs using a single DRL structure under various consumer preferences. In addition, through various case studies and comparative analysis, we evaluated the impact of different weather and driving patterns of the EV with different initial SOEs on the proposed algorithm. Furthermore, we verified that charging and discharging of the ESS and EV significantly contribute to the reduction of the cost of electricity.

The remainder of this paper is organized as follows. Section 2 introduces the various types of smart home appliances and the traditional HEMS optimization approach; it also provides an overview of the RL methodology. Section 3 presents the formulation of the proposed DRL-based HEMS algorithm based on the actor–critic method. The numerical examples for the proposed HEMS algorithm are reported in Section 4, and the conclusions are given in Section 5.

## 2. Background

### 2.1. Types of Smart Home Appliances

We consider the following types of smart home appliances in a single household where an HEMS automatically schedules their energy consumption under the time-of-use (TOU) pricing:

(1) Uncontrollable appliance ($A^{uc}$): An HEMS cannot manage the energy consumption scheduling of uncontrollable appliances such as televisions, personal computers, and lighting. Thus, an uncontrollable appliance is assumed to follow fixed energy consumption scheduling.
(2) Controllable appliance ($A^{c}$): It is an appliance for which the energy consumption scheduling is calculated by the HEMS. According to its operation characteristics, the controllable appliance is categorized into a reducible appliance ($A_{r}^{c}$) and shiftable appliance ($A_{s}^{c}$). A representative example of a reducible appliance is an air conditioner whose energy consumption can be curtailed to reduce the cost of electricity. By the contrast, under TOU pricing, the energy consumption scheduling of a shiftable appliance can be moved from one time slot to another to minimize the cost of electricity. A shiftable appliance has two types of load: (i) a non-interruptible load ($A_{s}^{c,NI}$), and (ii) an interruptible load ($A_{s}^{c,I}$). A shiftable appliance with an interruptible load can be interrupted at any time. For example, the HEMS must stop the discharging process and start the charging process of the ESS instantly when the PV power generation is greater than the load demand. However, the operation period of a shiftable appliance with a non-interruptible load must not be terminated by the HEMS. For example, a washing machine must finish a washing cycle prior to drying.

### 2.2. Traditional HEMS Optimization Approach

A conventional HEMS method that calculates the optimal operating scheduling of home appliances and DERs is formulated in terms of the following constrained multi-objective optimization problem described in the following section.

#### 2.2.1. Objective Function

The objective function (1) for the HEMS optimization problem comprises two terms, each of which includes different decision variables ($E_{t}^{\mathrm{net}},T_{t}^{\mathrm{in}}$) [25]:

(1) $$\min_{E_{t}^{\mathrm{net}},T_{t}^{\mathrm{in}}}\underset{J_{1}\left(E_{t}^{\mathrm{net}}\right)}{\underset{︸}{\sum_{t\in T}\pi_{t}E_{t}^{\mathrm{net}}}}+\underset{J_{2}\left(T_{t}^{\mathrm{in}}\right)}{\underset{︸}{\epsilon \sum_{t\in T}\left|T_{t}^{\mathrm{in}}-T^{\mathrm{set}}\right|}}.$$

The first term $J_{1}\left(E_{t}^{\mathrm{net}}\right)$ represents the total cost of electricity that is calculated under TOU pricing $\pi_{t}$ and $E_{t}^{\mathrm{net}}$, which is the net energy consumption accounting for the energy consumption of the controllable/uncontrollable appliances and the predicted PV generation output. The second term $J_{2}\left(T_{t}^{\mathrm{in}}\right)$ represents the total penalty related to the cost of the consumer’s discomfort. Here, discomfort is defined as a deviation of the consumer’s preferred temperature $T^{\mathrm{set}}$ from the indoor temperature $T_{t}^{\mathrm{in}}$. ϵ is a penalty for the cost of the consumer’s discomfort. A larger ϵ yields a smaller $J_{2}\left(T_{t}^{\mathrm{in}}\right)$, thereby offering decreasing discomfort to the consumer at the expense of less energy saving. The value of ϵ can be tuned by the HEMS operator to maintain the consumer’s preferred comfort level at the cost of a higher electricity bill. The equality and inequality constraints for the HEMS optimization problem are illustrated in the following subsections.

#### 2.2.2. Net Energy Consumption

Equation (2) expresses the constraint on the net energy consumption that represents the difference between the total consumption of all home appliances ($A=A_{r}^{c}⋃A_{s}^{c,NI}⋃A_{s}^{c,I}⋃A^{uc}$) and the predicted PV generation output $E_{t}^{\mathrm{PV}}$ at time *t*. In Equation (3), the total energy consumption of all appliances in Equation (2) is decomposed into four different types of consumptions corresponding to (i) reducible appliances ($a\in A_{r}^{c}$), (ii) shiftable appliances with a non-interruptible load ($a\in A_{s}^{c,NI}$), (iii) shiftable appliances with an interruptible load ($a\in A_{s}^{c,I}$), and (iv) uncontrollable appliances ($a\in A^{uc}$) [25]:

(2) $$E_{t}^{\mathrm{net}}=\sum_{a\in A}E_{a,t}-{\hat{E}}_{t}^{\mathrm{PV}}$$

(3) $$\begin{array}{cc} \sum_{a\in A}E_{a,t} & =\sum_{a\in A_{r}^{c}}E_{a,t}+\sum_{a\in A_{s}^{c,NI}}E_{a,t}\\ & +\sum_{a\in A_{s}^{c,I}}\left(E_{a,t}^{\mathrm{ch}}-E_{a,t}^{\mathrm{dch}}\right)+\sum_{a\in A^{uc}}E_{a,t}. \end{array}$$

#### 2.2.3. Operation Characteristics of Controllable Appliances

For a reducible appliance $a\in A_{r}^{c}$, (4) expresses the constraint for the indoor temperature dynamics of a reducible appliance (e.g., an AC) at time *t* ($T_{t}^{\mathrm{in}}$), which is expressed in terms of $T_{t-1}^{\mathrm{in}}$ at time t−1, the predicted outdoor temperature at time t−1 (${\hat{T}}_{t-1}^{\mathrm{out}}$), the energy consumption of the reducible appliances ($E_{a,t}$), and the environmental parameters (α,β) characterizing the indoor thermal condition [7]. Equation (5) illustrates the range of consumer’s preferred indoor temperatures. The energy consumption capacity for the reducible appliances is limited according to (6):

(4) $$\begin{array}{cc} T_{t}^{\mathrm{in}} & =T_{t-1}^{\mathrm{in}}+\alpha \left({\hat{T}}_{t-1}^{\mathrm{out}}-T_{t-1}^{\mathrm{in}}\right)+\beta E_{a,t} \end{array}$$

(5) $$\begin{array}{cc} T^{\min} & \leq T_{t}^{\mathrm{in}}\leq T^{\max} \end{array}$$

(6) $$\begin{array}{cc} E_{a}^{\min} & \leq E_{a,t}\leq E_{a}^{\max}. \end{array}$$

Equations (7)–(9) guarantee the consumer’s preferred operation of shiftable appliances with a non-interruptible load $a\in A_{s}^{c,NI}$ (e.g., a WM) with the binary decision variable $b_{a,t}^{c,NI}$ in different situations: (i) for a stopping period in which $\omega_{s}^{\mathrm{pref}}$ and $\omega_{f}^{\mathrm{pref}}$ are the consumer’s preferred starting and finishing time (7), respectively; (ii) for an operation period of $L_{a}$ hours during a day in (8); and (iii) for a consecutive operation period of $L_{a}$ hours in (9). The energy consumption capacity for the shiftable appliances with a non-interruptible load is calculated using (10):

(7) $$\begin{array}{cc} b_{a,t}^{c,NI} & =0,\;\;\;\;\;\;\;\;\;\;t\in \left[1,\omega_{s}^{\mathrm{pref}}\right)\cup \left(\omega_{f}^{\mathrm{pref}},T\right] \end{array}$$

(8) $$\begin{array}{cc} \sum_{t=\omega_{s}^{\mathrm{pref}}}^{\omega_{f}^{\mathrm{pref}}}b_{a,t}^{c,NI} & =L_{a} \end{array}$$

(9) $$\sum_{t=p}^{p+L_{a}-1}b_{a,t}^{c,NI}\geq \left(b_{p}^{c,NI}-b_{p-1}^{c,NI}\right)L_{a},\forall p\in (\omega_{s}^{\mathrm{pref}},\omega_{f}^{\mathrm{pref}}-L_{a}+1)$$

(10) $$\begin{array}{cc} E_{a,t} & =b_{a,t}^{c,NI}E_{a}^{\max}. \end{array}$$

Equation (11) presents the operational dynamics of the SOE for the ESS and EV ($a\in A_{s}^{c,I}$) at current time instant *t* in terms of the SOE at previous time instant *t*-1, the charging and discharging efficiency, i.e., $\eta_{a}^{\mathrm{ch}}$ and $\eta_{a}^{\mathrm{dch}}$, and the charging and discharging energy, i.e., $E_{a,t}^{\mathrm{ch}}$ and $E_{a,t}^{\mathrm{dch}}$, respectively [11]. Equation (12) represents the SOE capacity constraint for the ESS and EV. Equations (13) and (14) present the limits of the charging ($E_{a,t}^{\mathrm{ch}}$) and discharging ($E_{a,t}^{\mathrm{dch}}$) energies of the ESS and EV, respectively, where $b_{a,t}^{c,I}$ represents the binary decision variable that determines the charging and discharging status of the ESS and EV:

(11) $$\begin{array}{cc} SOE_{a,t}=SOE_{a,t-1} & +\eta_{a}^{\mathrm{ch}}E_{a,t}^{\mathrm{ch}}-\frac{E_{a,t}^{\mathrm{dch}}}{\eta_{a}^{\mathrm{dch}}} \end{array}$$

(12) $$\begin{array}{cc} SOE_{a}^{\min}\leq & SOE_{a,t}\leq SOE_{a}^{\max} \end{array}$$

(13) $$\begin{array}{cc} E_{a}^{\mathrm{ch},\min}b_{a,t}^{c,I}\leq & E_{a,t}^{\mathrm{ch}}\leq E_{a}^{\mathrm{ch},\max}b_{a,t}^{c,I} \end{array}$$

(14) $$\begin{array}{cc} E_{a}^{\mathrm{dch},\min}\left(1-b_{a,t}^{c,I}\right)\leq & E_{a,t}^{\mathrm{dch}}\leq E_{a}^{\mathrm{dch},\max}\left(1-b_{a,t}^{c,I}\right). \end{array}$$

Notably, the constraints (11)–(14) for the EV remain true in $t\in [\omega^{\mathrm{arr}},\omega^{\mathrm{dep}}]$, whereas $E_{a,t}^{\mathrm{ch}}$ and $E_{a,t}^{\mathrm{dch}}$ become zero in $t\notin [\omega^{\mathrm{arr}},\omega^{\mathrm{dep}}]$. In addition, when the EV departs from home at $t=\omega^{\mathrm{dep}}$, the SOE of the EV must be larger than the consumer preferred SOE $SOE_{a}^{\mathrm{pref}}$

(15) $$SOE_{a,t}\geq SOE_{a}^{\mathrm{pref}}.$$

### 2.3. Reinforcement Learning Methodology

#### 2.3.1. Reinforcement Learning

RL is an ML method that addresses a problem in a specific environment with the objective of maximizing a numerical reward. This learning process is applied to various types of general and special engineering problems. In the RL framework, while an agent interacts with an environment, it learns a particular type of action depending on the state of the environment and conveys the learned action to the environment. The environment then returns a reward along with its new state to the agent. This learning process continues until the agent maximizes the total cumulative rewards received from the environment.

A policy is defined in terms of the procedure through which the agent acts from a specific state. The main objective of the agent is to find an optimal policy that maximizes the agent’s cumulative reward in the environment. In our study, we consider that the environment is characterized by a Markov decision process, in which the change of the agent’s next state depends only on the current state, along with the action chosen in the current state ignoring all previous states and actions.

In this study, the value function is selected as $Q(s_{t},a_{t})$, namely the Q-value, which is written in terms of a pair of state $s_{t}$ and action $a_{t}$ at a discrete time *t*. By using the Q-value, the agent’s main objective is to achieve the maximum Q-value at every time step *t*. Q-learning is one of the basic RL methods to find the optimal policy $\nu^{*}$ in decision-making problems. The general *Q*-learning process computes and updates the Q-value $Q(s_{t},a_{t})$ to achieve the maximum total rewards using the following Bellman equation:

(16) $$Q_{\nu^{*}}^{*}\left(s_{t},a_{t}\right)=r\left(s_{t},a_{t}\right)+\gamma \max Q\left(s_{t+1},a_{t+1}\right)$$

where, based on the optimal policy $\nu^{*}$, the optimal Q-value $Q_{\nu^{*}}^{*}\left(s_{t},a_{t}\right)$ is obtained by the summation of the present reward $r(s_{t},a_{t})$ and the maximum discounted future reward $\gamma \max Q(s_{t+1},a_{t+1})$.

In general, a discounting factor γ∈[0,1] is used to explain the relative importance of the current and future rewards. As the discounting factor γ decreases, the agent becomes short-sighted because it increasingly focuses on the current reward. However, a larger γ enables the agent to focus increasingly on the future reward and thus becomes far-sighted. The value of γ can be tuned by the system operator to balance the current and future rewards.

#### 2.3.2. Actor–Critic Method

The actor–critic method is an extension of the policy gradient method that can improve the stability and reduce the variance of the gradient when the optimal solution of the algorithm converges [33]. If the value of a certain state of the agent is known, the corresponding Q-value can be calculated and applied to the REINFORCE method (Algorithm 1) to calculate the gradient of policy network parameters and renew the agent’s policy network, thereby leading to a better cumulative result by increasing the probability for the agent’s action. In the actor–critic method, the agent can use an additional network to judge the goodness of the action the agent selects in a certain state. The policy network that returns the probability of the agent’s action is called actor network, whereas the network that returns the evaluation value of the agent’s action is called a critic network. The policy gradient method is suitable for handling the problem with a continuous action space; however, this method may have a poor convergence performance. An additional critic network in the actor–critic method can resolve the convergence issue in the policy gradient method. In our study, the actor and critic networks share a common body network owing to an effective convergence consideration.

The actor network returns the probability of the action that the agent selects in a particular state and the critic network returns the numerical future value that the agent would obtain in the terminal state. The critic network updates the function that distinguishes between the action and value, whereas the policy network updates its parameters in the direction suggested by the critic network. In this study, the parameters in the actor network are updated by the REINFORCE method (Algorithm 1) and the parameters in the critic network are updated by a linear temporal difference (TD) method [34]. The TD method directly learns from episodes of experience, and the present and guessed values. This is known to be a useful method to solve the Markov decision process (MDP) problem. In our study, we select the linear value function approximation for applying the TD method to the critic network. The actor–critic method updates the parameters of the actor and critic networks to minimize the TD error, which encodes the difference between the value function of the present state and target value function. Algorithm 2 illustrates the actor–critic approach with the TD method. In Algorithm 2, *∂*, θ, and ω represent the TD error from the Q-value, the parameter of the actor network, and the parameter of the critic network, respectively. $\xi_{\theta }$ and $\xi_{\omega }$ are the learning rates of the actor network and critic networks in the algorithm, respectively.
**Algorithm 1:** REINFORCE method
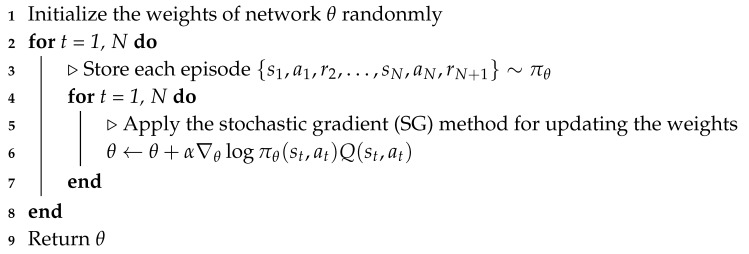


**Algorithm 2:** Actor-critic method

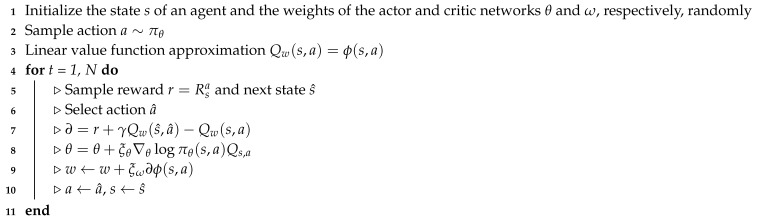



## 3. Proposed Method for DRL-Based Home Energy Management

In this section, we propose a hierarchical two-level DRL framework that employs the actor–critic method to schedule the day-ahead optimal energy consumption of a single household with smart home appliances and DERs within the consumer’s preferred appliance scheduling and comfort range. In the proposed framework, the energy consumption scheduling problem is decomposed into a two-level DRL problem corresponding to the energy consumption scheduling of i) WM and AC at the first level and ii) ESS and EV at the second level. A detailed illustration of the state, action, and reward for each level of the proposed actor–critic method is provided in the following two subsections. In addition, the superscript of the variables for the state/action spaces and the reward function represents the first and second level, respectively.

### 3.1. Energy Management Model for WM and AC: Level 1

#### 3.1.1. State Space

We consider the situation in which the proposed DRL-based HEMS algorithm performs optimal day-ahead scheduling of appliances with a 1-h scheduling resolution. For ∀t=1,…,24, the state space of the agent for WM and AC at the first level is defined as follows:

(17) $$S^{\left(1\right)}=\left\{t,\pi_{t},{\hat{T}}_{t}^{\mathrm{out}},T_{t-1}^{\mathrm{in}}\right\}$$

where the states *t*, $\pi_{t}$, ${\hat{T}}_{t}^{\mathrm{out}}$, and $T_{t-1}^{\mathrm{in}}$ denote the scheduling time of the WM and AC, TOU price, and outdoor and indoor temperatures, respectively, at time *t*.

#### 3.1.2. Action Space

The optimal action for the first level relies on the environment of the agent including the present state, as defined in Section 3.1.1. The action space at the first level is defined as follows:

(18) $$A^{\left(1\right)}=\left\{E_{t}^{\mathrm{WM}},E_{t}^{\mathrm{AC}}\right\}$$

where $E_{t}^{\mathrm{WM}}$ and $E_{t}^{\mathrm{AC}}$ represent the energy consumption of the WM and AC at time *t*, respectively. In this study, $E_{t}^{\mathrm{AC}}$ has a continuous value, whereas $E_{t}^{\mathrm{WM}}$ has a discrete value; $E_{t}^{\mathrm{WM}}=E^{\mathrm{WM},\max}$ when the WM turns on, otherwise, $E_{t}^{\mathrm{WM}}=0$.

#### 3.1.3. Reward

The reward function for the first level is formulated as the sum of the negative cost of electricity and dissatisfaction of WM and AC related to the consumer’s preferred comfort and appliance’s operation characteristics. The total reward at the first level is expressed as:

(19) $$R_{t}^{\left(1\right)}=-\left(c_{t}^{\mathrm{WM}}+c_{t}^{\mathrm{AC}}\right)$$

where $c_{t}^{\mathrm{WM}}$ and $c_{t}^{\mathrm{AC}}$ are the cost functions for the WM and AC, respectively. Each cost function includes the cost of electricity for the appliance along with the cost of the consumer’s dissatisfaction for the undesired operation of the WM and the indoor thermal discomfort.

First, the cost function of the WM is defined as

(20) $$\begin{array}{c} c_{t}^{\mathrm{WM}}=\left\{\begin{array}{c} \pi_{t}E_{t}^{\mathrm{WM}}+\bar{\delta }\left(\omega_{s}^{\mathrm{pref}}-t\right),\;\;\;\;\;\mathrm{if}\;t<\omega_{s}^{\mathrm{pref}}\\ \pi_{t}E_{t}^{\mathrm{WM}}+\underline{\delta }\left(t-\omega_{f}^{\mathrm{pref}}\right),\;\;\;\;\;\mathrm{if}\;t>\omega_{f}^{\mathrm{pref}}\\ \pi_{t}E_{t}^{\mathrm{WM}},\;\;\;\;\;\;\;\;\;\;\;\;\;\;\;\;\;\;\;\;\;\;\;\;\;\mathrm{otherwise} \end{array}\right. \end{array}$$

where $\omega_{s}^{\mathrm{pref}}$ and $\omega_{f}^{\mathrm{pref}}$ are the consumer’s preferred starting and finishing times of the WM, respectively, while $\bar{\delta }$ and $\underline{\delta }$ are the penalties for early and late operations, respectively, compared to the consumer’s preferred operation interval. The cost of dissatisfaction is added to the cost function if the WM schedules the WM energy consumption earlier than $\omega_{s}^{\mathrm{pref}}$ or later than $\omega_{f}^{\mathrm{pref}}$; otherwise, the cost function includes only the cost of electricity.

The cost function of the AC is expressed as:

(21) $$\begin{array}{c} c_{t}^{\mathrm{AC}}=\left\{\begin{array}{c} \pi_{t}E_{t}^{\mathrm{AC}}+\bar{\kappa }\left(T^{\min}-T_{t}^{\mathrm{in}}\right),\;\;\;\;\mathrm{if}\;T_{t}^{\mathrm{in}}<T^{\min}\\ \pi_{t}E_{t}^{\mathrm{AC}}+\underline{\kappa }\left(T_{t}^{\mathrm{in}}-T^{\max}\right),\;\;\;\;\mathrm{if}\;T_{t}^{\mathrm{in}}>T^{\max}\\ \pi_{t}E_{t}^{\mathrm{AC}},\;\;\;\;\;\;\;\;\;\;\;\;\;\;\;\;\;\;\;\;\;\;\;\;\;\;\;\;\;\;\;\;\mathrm{otherwise} \end{array}\right. \end{array}$$

where $\bar{\kappa }$ and $\underline{\kappa }$ are the penalties for the consumer’s thermal discomfort. The cost of dissatisfaction is defined as the deviation of the consumer’s preferred temperature $T_{t}^{\mathrm{in}}$ from $T^{\min}$ and $T^{\max}$.

It is noted that two terms in (20) and (21) have a trade-off relationship between the saving of the electricity cost and the reduction of the consumer’s dissatisfaction cost in terms of the penalties $\left\{\bar{\delta },\underline{\delta }\right\}$ and $\left\{\bar{\kappa },\underline{\kappa }\right\}$, respectively. On the trade-off relationship, HEMS operators using our proposed DRL algorithm can adaptively adjust and tune the penalty to the situations where the consumer aims to save the electricity cost more or maintain the consumer’s desired comfort and preference. The selection of the values of these penalties would become different depending on the consumer’s desired comfort level and environment.

### 3.2. Energy Management Model for ESS and EV: Level 2

The optimal schedules of the energy consumption of the WM and AC from the first level along with the fixed load of the uncontrollable appliances are embedded into the actor–critic module at the second level. In the second level, the agent for the ESS and EV initiates the learning process to determine the optimal charging and discharging schedules of the ESS and EV to minimize the cost of electricity. During the learning process in this second level, the energy generated by the PV system is assumed to be charged first to the ESS; then, the ESS will select an appropriate action.

#### 3.2.1. State Space

The state space of the agent at the second level, which manages the operations of the ESS and EV, is defined as

(22) $$S^{\left(2\right)}=\left\{t,\pi_{t},SOE_{t}^{\mathrm{ESS}},SOE_{t}^{\mathrm{EV}},{\hat{E}}_{t}^{\mathrm{PV}},E_{t}^{\left(1\right)}\right\}$$

where the states *t*, $\pi_{t}$, $SOE_{t}^{\mathrm{ESS}}$, $SOE_{t}^{\mathrm{EV}}$, ${\hat{E}}_{t}^{\mathrm{PV}}$, and $E_{t}^{\left(1\right)}$ are the scheduling time of the ESS and EV, the TOU price, SOE of the ESS and EV, the predicted PV generation output, and the aggregated energy consumption schedule calculated at the first level, respectively, at time *t*.

#### 3.2.2. Action Space

Similar to the action space of the WM and AC in Section 3.1.2, the action space of the ESS and EV at the second level is expressed as

(23) $$A^{\left(2\right)}=\left\{E_{t}^{\mathrm{ESS}},E_{t}^{\mathrm{EV}}\right\}$$

where $E_{t}^{\mathrm{ESS}}$ and $E_{t}^{\mathrm{EV}}$ represent the continuous energy charging and discharging of the ESS and EV, respectively, at time *t*.

Note that, in the proposed two-level DRL architecture, the agent for the ESS and EV selects their optimal charging and discharging action using $E_{t}^{\left(1\right)}$ (22) that includes the action of the agent for the WM and AC along with the fixed load of the uncontrollable appliances at the first level. If the DRL-based HEMS algorithm is modelled as a single-level framework (i.e., the state spaces (17), (22) and the action spaces (18), (23) at the first and second levels are combined, respectively), the agent for the ESS and EV may not find its optimal policy because the ESS and EV have no consumption data of other appliances in their state space. This is verified in Section 4.2.

#### 3.2.3. Reward

The reward for the second level is formulated as the sum of the negative cost of electricity and dissatisfaction of ESS and EV associated with the consumer’s preferred comfort and appliance’s operation characteristics. The total reward at the second level is defined as

(24) $$R_{t}^{\left(2\right)}=-\left(c_{t}^{\mathrm{ESS}}+c_{t}^{\mathrm{EV}}\right).$$

In (24), $c_{t}^{\mathrm{ESS}}$ and $c_{t}^{\mathrm{EV}}$ represent the cost functions for the ESS and EV, respectively. Each cost function includes the cost of electricity of the appliance along with the cost of dissatisfaction for underdischarging and overcharging of the ESS and EV. Notably, these cost functions include the discharging energy from the ESS and EV, which supports the uncovered energy consumption of aggregated load for WM, AC, and uncontrollable appliances.

First, the cost function of the ESS is expressed as follows:

(25) $$\begin{array}{c} c_{t}^{\mathrm{ESS}}=\left\{\begin{array}{c} \pi_{t}E_{t}^{\mathrm{ESS}}+\bar{\tau }\left(SOE_{t}^{\mathrm{ESS}}-SOE^{\mathrm{ESS},\max}\right),\\ \;\;\;\;\;\;\;\;\;\;\;\;\;\;\;\;\;\;\;\;\;\;\;\;\;\;\;\;\;\mathrm{if}\;SOE_{t}^{\mathrm{ESS}}>SOE^{\mathrm{ESS},\max}\\ \pi_{t}E_{t}^{\mathrm{ESS}}+\underline{\tau }\left(SOE^{\mathrm{ESS},\min}-SOE_{t}^{\mathrm{ESS}}\right),\\ \;\;\;\;\;\;\;\;\;\;\;\;\;\;\;\;\;\;\;\;\;\;\;\;\;\;\;\;\;\mathrm{if}\;SOE_{t}^{\mathrm{ESS}}<SOE^{\mathrm{ESS},\min}\\ \pi_{t}E_{t}^{\mathrm{ESS}},\;\;\;\;\;\;\;\;\;\;\;\;\;\;\;\;\;\;\mathrm{otherwise}, \end{array}\right. \end{array}$$

where $\bar{\tau }$ and $\underline{\tau }$ are the penalties for ESS overcharging and undercharging, respectively. In this case, energy underutilization and dissipation of the ESS occur if the SOE becomes lower than $SOE^{\min}$ (undercharging) or greater than $SOE^{\max}$ (overcharging).

Next, the cost function of the EV is expressed as

(26) $$\begin{array}{c} c_{t}^{\mathrm{EV}}=\left\{\begin{array}{c} \pi_{t}E_{t}^{\mathrm{EV}}+\bar{\nu }\left(SOE_{t}^{\mathrm{EV}}-SOE^{\mathrm{EV},\max}\right),\\ \;\;\;\;\;\;\;\;\;\;\;\;\mathrm{if}\;SOE_{t}^{\mathrm{EV}}>SOE^{\mathrm{EV},\max},\;\;t\in \left[\omega^{\mathrm{arr}},\omega^{\mathrm{dep}}\right]\\ \pi_{t}E_{t}^{\mathrm{EV}}+\underline{\nu }\left(SOE^{\mathrm{EV},\min}-SOE_{t}^{\mathrm{EV}}\right),\\ \;\;\;\;\;\;\;\;\;\;\;\;\mathrm{if}\;SOE_{t}^{\mathrm{EV}}<SOE^{\mathrm{EV},\min},\;\;t\in \left[\omega^{\mathrm{arr}},\omega^{\mathrm{dep}}\right]\\ \pi_{t}E_{t}^{\mathrm{EV}}+\underline{\eta }\left(SOE^{\mathrm{pref}}-SOE_{t}^{\mathrm{EV}}\right),\\ \;\;\;\;\;\;\;\;\;\;\;\;\mathrm{if}\;t=\omega^{\mathrm{dep}}\;\mathrm{and}\;SOE_{t}^{\mathrm{EV}}<SOE^{\mathrm{pref}}\\ \pi_{t}E_{t}^{\mathrm{EV}},\;\;\;\;\;\;\;\;\mathrm{otherwise}, \end{array}\right. \end{array}$$

where $\bar{\nu }$ and $\underline{\nu }$ are the penalties for overcharging and undercharging of the EV, respectively. Similar to the operation of the ESS, energy underutilization and dissipation occur if the SOE of the EV becomes lower than $SOE^{\mathrm{EV},\min}$ or higher than $SOE^{\mathrm{EV},\max}$. Unlike the reward function of the ESS, the reward function of the EV includes the parameter $\underline{\eta }$, which denote the consumer’s preference penalty of the EV, corresponding to the deviation of the SOE of the EV from the consumer’s preferred SOE when the EV departs. If $SOE_{t}^{\mathrm{EV}}$ is lower than $SOE^{\mathrm{pref}}$ at departure time $\omega^{\mathrm{dep}}$, the cost of dissatisfaction increases owing to insufficient SOE.

### 3.3. Proposed Actor–Critic-Based HEMS Algorithm

**Figure 2:**
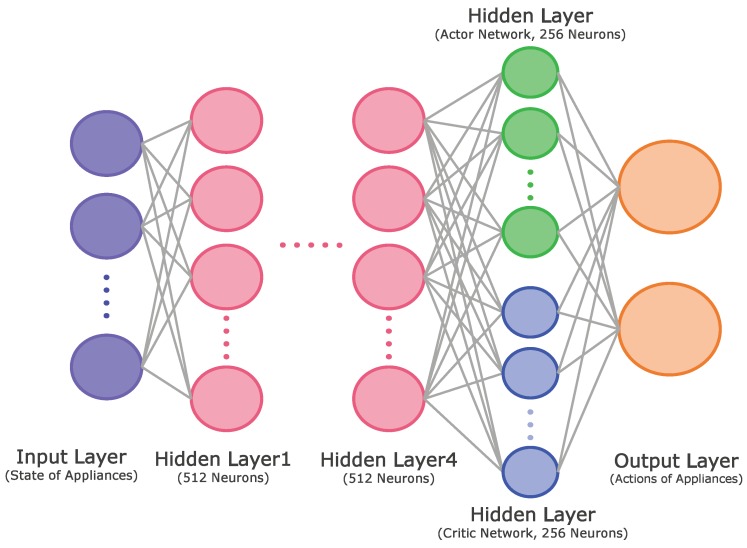
Architecture of the neural network model for the proposed actor–critic method.

In this subsection, we illustrate the proposed DRL method based on the actor–critic method that determines the optimal policy to minimize the electricity bill within the consumer’s preferred comfort level and the appliance operation characteristics. Compared to value-based RL methods, the policy gradient approach is appropriate for engineering problems with continuous action spaces. In general, the continuous policy gradient network obtains state information from the agent and returns the appropriate action using a normal distribution. The network yields the mean and variance to achieve a normal distribution, and the agent samples the action randomly based on the resulting distribution. In the actor–critic approach, the additory method of criticizing the Q-value for its efficiency and convergence is added. Therefore, the network provides the mean, variance, and Q-values to find the optimal actions. As shown in Figure 2, the proposed actor–critic network model for each level consists of one input layer for state elements, four hidden layers for a common body network with 512 neurons, one hidden layer for each actor and critic networks with 256 neurons, and one output layer with means and variances of the operation schedules of appliances and Q-values. In this study, a hyperbolic tangent function was used as a transfer function. In addition, the adaptive moment estimation (ADAM) optimization algorithm [35] was used for training the proposed DRL model with a learning rate of 0.00004. Finally, Algorithm 3 illustrates the procedure of the actor–critic-based HEMS algorithm that learns the energy management policies, which optimize the cost of electricity and consumer’s comfort level for level 1 (WM and AC) and level 2 (ESS and EV).
**Algorithm 3:** Proposed actor–critic-based energy management of smart home at level 1 (or level 2).
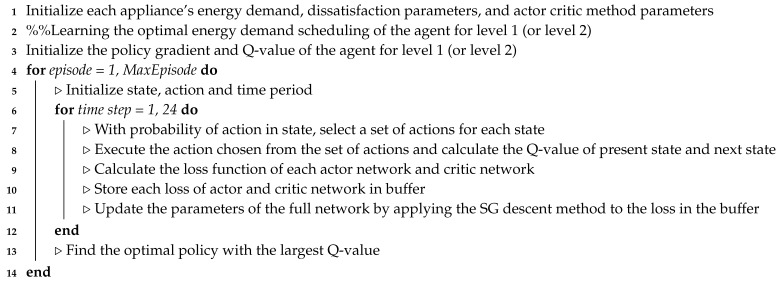


## 4. Numerical Examples

### 4.1. Simulation Setup

**Figure 3:**
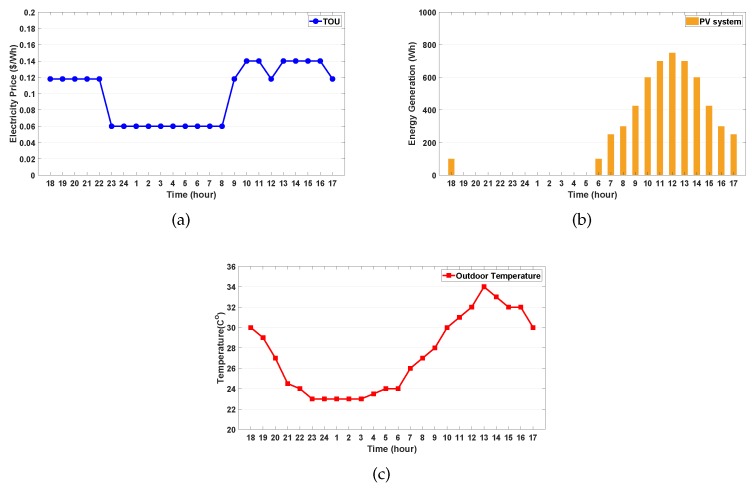
Profiles of electricity price and weather. (**a**) time-of-use (TOU) price; (**b**) photovoltaic (PV) generation; (**c**) outdoor temperature.

Under the TOU pricing shown in Figure 3a, we considered a household where the proposed DRL algorithm schedules the operation of two tasks: (i) two major controllable home appliances (WM and AC) at the first level and (ii) controllable DERs (PV-integrated ESS and EV) at the second level. The simulations were executed for 24 h with a 1-h scheduling resolution.

We assumed that the predicted PV generation energy ${\hat{E}}_{t}^{\mathrm{PV}}$ in Figure 3b and predicted outdoor temperature ${\hat{T}}_{t}^{\mathrm{out}}$ in Figure 3c could be calculated accurately. The maximum energy consumptions of the WM and AC were set to 300 and 1000 Wh, respectively. For the ESS, the battery capacity is 4000 Wh, and the minimum ($SOE^{\mathrm{ESS},\min}$), maximum ($SOE^{\mathrm{ESS},\max}$) and initial SOE ($SOE_{\mathrm{ini}}^{\mathrm{ESS}}$) of the ESS were set to 400 Wh (10% $SOE^{\mathrm{ESS},\max}$), 4000 Wh (100% $SOE^{\mathrm{ESS},\max}$), and 2000 Wh (50% $SOE^{\mathrm{ESS},\max}$), respectively. The maximum charging and discharging energy of the ESS were both 1200 Wh. For the EV, the battery capacity was 17,000 Wh, and the minimum ($SOE^{\mathrm{EV},\min}$), maximum ($SOE^{\mathrm{EV},\max}$) and initial SOE ($SOE_{\mathrm{ini}}^{\mathrm{EV}}$) of the EV were set to 1700 Wh (10% $SOE^{\mathrm{EV},\max}$), 17,000 Wh (100% $SOE^{\mathrm{EV},\max}$), and 9350 Wh (55% $SOE^{\mathrm{EV},\max}$), respectively. The maximum charging and discharging energy of the EV were both 10,000 Wh. For the reward function, the consumer’s preferred operating period $\left[\omega_{s}^{\mathrm{pref}},\omega_{f}^{\mathrm{pref}}\right]$ for the WM was set to [9:00 a.m., 10:00 p.m.] along with 2 h of consecutive operation time. The range of consumer’s comfortable indoor temperature $\left[T^{\min},T^{\max}\right]$ controlled by the AC was set to [22.5 °C, 25.5 °C]. For the EV, the preferred SOE ($SOE^{\mathrm{pref}}$) and the departure time ($\omega^{\mathrm{dep}}$) of the EV were set to 12,750 Wh (75% $SOE^{\mathrm{EV},\max}$) and 8:00 a.m., respectively. The pairs of penalties for the cost of dissatisfaction of the WM, AC, ESS, and EV were {$\left(\bar{\delta }=50,\underline{\delta }=50\right)$, $\left(\bar{\kappa }=200,\underline{\kappa }=200\right)$, $\left(\bar{\tau }=100,\underline{\tau }=100\right)$, $\left(\bar{\nu }=100,\underline{\nu }=100\right)$, and $\left(\underline{\eta }=50\right)$}, respectively.

The performance of the proposed approach was tested for the following four cases according to different weather, weekday/weekend, and initial SOE $SOE_{\mathrm{ini}}^{\mathrm{EV}}$ of the EV:

- Case 1: Sunny, weekday, $SOE_{\mathrm{ini}}^{\mathrm{EV}}=0.55\times SOE^{\mathrm{EV},\max},$
- Case 2: Rainy, weekday, $SOE_{\mathrm{ini}}^{\mathrm{EV}}=0.55\times SOE^{\mathrm{EV},\max},$
- Case 3: Sunny, weekend, $SOE_{\mathrm{ini}}^{\mathrm{EV}}=0.55\times SOE^{\mathrm{EV},\max},$
- Case 4: Sunny, weekday, $SOE_{\mathrm{ini}}^{\mathrm{EV}}=0.15\times SOE^{\mathrm{EV},\max}.$

On a sunny day, the predicted PV generation output at time *t* (${\hat{E}}_{t}^{\mathrm{PV}}$) follows the profile in Figure 3b, and on a rainy day, it is set to zero. On a weekday, the EV is assumed to arrive at the household at 6:00 p.m. and then the charging and discharging processes are conducted until the EV departs from the household at 8:00 a.m. During the weekend, the EV charges or discharges energy during 24 h. $SOE_{\mathrm{ini}}^{\mathrm{EV}}$ denotes the SOE when the EV arrives at home, and different values of $SOE_{\mathrm{ini}}^{\mathrm{EV}}$ represent different driving distances of the EV. All the cases were tested using Python 3.7.0 with the machine learning package pytorch 1.1.0.

### 4.2. Simulation Results at Level 1

**Figure 4:**
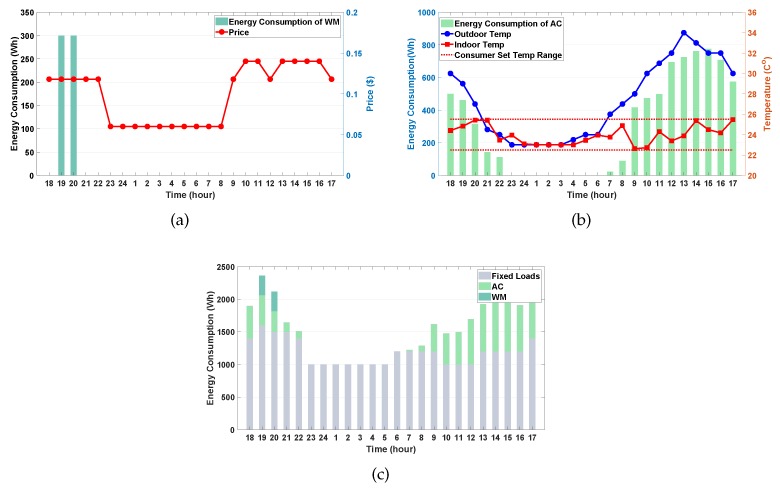
DRL-based energy consumption schedule. (**a**) washing machine (WM); (**b**) air conditioner (AC); (**c**) WM+AC+Uncontrollable appliances at Level 1.

In this subsection, we report the simulation results of the proposed approach associated with level 1 and verify the optimal energy consumption schedule of the WM and AC along with the consumer’s comfort level. Figure 4a shows the energy consumption schedule of the WM. We observe from Figure 4a that the operation period is selected as [7:00 p.m., 8:00 p.m.] with two consecutive operation hours. This scheduling policy is optimal because the WM operates at the lowest TOU pricing during the consumer’s preferred operation period [9:00 a.m., 10:00 p.m.], which in turn reduces the electricity bill while satisfying the consumer’s preference and operation characteristics of the WM. Figure 4b illustrates the energy consumption schedule of the AC. Unlike the observation in Figure 4a, the AC energy consumption is scheduled at an even higher TOU pricing during the period [12:00 p.m., 4:00 p.m.]. This was expected because the agent at the first level considers the consumer’s thermal comfort as well as saving on electricity bills in the reward function. As shown in Figure 4b, the AC turns off at midnight when the outdoor temperature is within the range of consumer’s preferred temperatures. When an indoor temperature violation occurs at 7:00 a.m. owing to a sharp increase of the outdoor temperature, the AC turns on and its energy consumption increases to maintain the consumer’s preferred indoor temperature. The maximum energy consumption schedule is verified during the interval [12:00 p.m., 4:00 p.m.], which presents the highest TOU pricing. In this interval, the AC aims to satisfy the consumer’s comfort at the expense of increased electricity bills. Figure 4c shows the total energy consumption schedule of the WM, AC, and uncontrollable appliances with fixed loads at level 1. Note that the sum of energy consumption schedules of WM, AC, and uncontrollable appliances at each period is used by the actor–critic module at the second level, which in turn determines the optimal policy of charging and discharging for the ESS and EV.

### 4.3. Simulation Results at Level 2

In this subsection, we present the simulation results of the proposed approach at Level 2. These results are divided into three comparison tests using four cases described in Section 4.1: {Case 1, Case 2}, {Case 1, Case 3}, and {Case 1, Case 4}. The discharging ratio of the EV and ESS to cover the energy consumption at the first level simultaneously was set to 0.8 and 0.2, respectively. This ratio was determined as the battery capacity of the EV divided by the battery capacity of the ESS.

#### 4.3.1. Case 1 vs. Case 2

**Figure 5:**
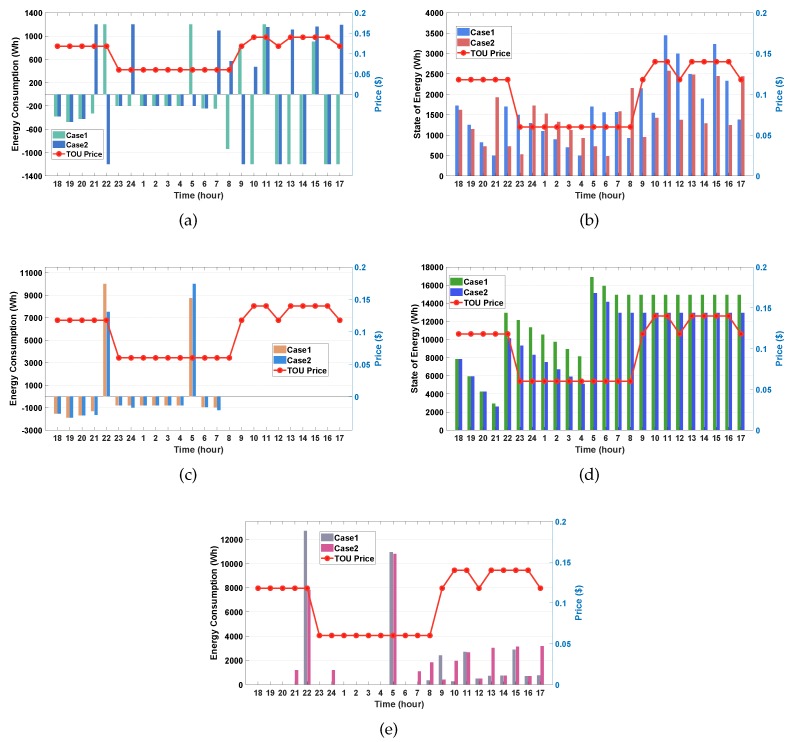
Performance comparison between Case 1 and Case 2. (**a**) energy consumption of the energy storage system (ESS); (**b**) state of energy (SOE) of the ESS; (**c**) energy consumption of the electric vehicle (EV); (**d**) SOE of the EV; (**e**) net consumption of household.

In this simulation, we investigate and compare the performance between Case 1 (with the PV generation output) and Case 2 (without the PV generation output). Figure 5a,b show the charging/discharging and SOE schedules of the ESS for Case 1 and Case 2, respectively. We observe from Figure 5a that, in general, more discharging (negative energy consumption) of the ESS for both cases occurs at high TOU pricing to support the household energy demand, thereby leading to consumer’s energy savings. In addition, it can be observed through the comparison of Figure 5a,b that the SOE of the ESS increases (or decreases) as the ESS charges (or discharges) energy. However, Case 1 shows an unexpected phenomenon where the SOE of the ESS in the scheduling period between 10:00 a.m and 4:00 p.m is higher than in other periods even though the ESS conducts a significant energy discharging in this period. This phenomenon occurs because the PV generation energy is injected into the ESS further than the ESS discharging energy in this period.

However, we observe from Figure 5a,b that the ESS charges more power at high TOU pricing for Case 2 than for Case 1. This observation justifies that the different weather conditions influence the charing and discharging schedules of the ESS significantly.

Figure 5c,d show the charging/discharging and SOE schedules for the EV. We observe from these figures that the EVs for Cases 1 and 2 does not perform neither charging nor discharging processes after 7:00 a.m. and that the SOE level remains unchanged, respectively. This observation is consistent with the EVs departure time setting ($\omega^{\mathrm{dep}}$=8:00 a.m.). We also observe from Figure 5c that the EVs charge significantly large amounts of energy from the grid at 10:00 p.m. and 5:00 a.m., whereas it discharges energy to support the household energy demand in the other time periods. This charging process derives from the fact that the EVs charge sufficient energy in advance to satisfy the consumer’s preferred SOE condition ($SOE^{\mathrm{pref}}$=12,750 Wh). It can be verified from Figure 5d that the SOE at 8:00 a.m. exceeds 12,750 Wh for both cases. However, no unexpected phenomenon observed in Figure 5a,b is identified in Figure 5c,d. This is because the PV generation output affects only the ESS charging and discharging.

Figure 5e shows the net energy consumptions of the household for Cases 1 and 2, which is the difference between the energy consumption of the controllable/uncontrollable appliances and the predicted PV generation output. As shown in this figure, the values of the net energy consumption of both cases are much larger at 10:00 p.m. and 5:00 a.m. than at the other time slots owing to the large EV charging at these two time slots. However, in the period between 8:00 a.m. and 5:00 p.m., we can identify the large amount of energy consumption in Case 2. This is because the ESS needs to charge more power in Case 2 owing to no PV generation than in Case 1.

#### 4.3.2. Case 1 vs. Case 3

**Figure 6:**
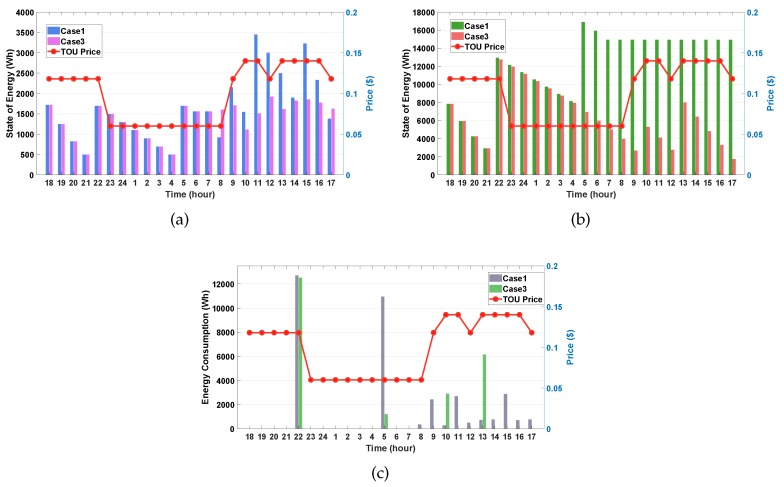
Performance comparison between Case 1 and Case 3. (**a**) SOE of the ESS; (**b**) SOE of the EV; (**c**) net consumption of household.

In this case study, we investigate and compare the performance between Case 1 (on a weekday) and Case 3 (on a weekend). We considered that the EV stays at home during 24 h in the weekend. Given that the EV does not depart, the consumer’s preferred SOE of the EV is not considered in the proposed algorithm. First, we can observe from Figure 6a that the SOE of the ESS in Case 3 is generally lower than that in Case1 even if the weather is sunny. We can interpret this observation as follows.

In Case 3, the ESS does not need to allocate much stored energy to satisfy the consumer’s preferred SOE of the EV because the EV stays at home all day. Thus, the ESS increasingly supports the household energy demand through its charging process along with the EV charging. Unlike the results for Cases 1 and 2, we can verify from Case 3 in Figure 6b that the consumer’s preferred SOE of EV condition is ignored during the scheduling process of EV energy consumption, in which the SOE of the EV at 8:00 a.m. is much lower than $SOE^{\mathrm{pref}}$ = 12,750 Wh. After 8:00 a.m., the EV keeps charging and discharging the energy in the same way as the ESS. We also observe from Figure 6c that in Case 3 a high net energy consumption occurs at 10:00 p.m. and 1:00 p.m. owing to EV charging for discharging plan in future scheduling. Moreover, the net energy consumption at 5:00 a.m. in Case 3 is much smaller than in Case 1 because the consumer’s preferred SOE of EV is ignored. Note from Case 3 in Figure 6c that zero-energy consumption is verified in the period between 8:00 a.m. and 5:00 p.m. except 10:00 a.m. and 1:00p.m. This result shows that more energy saving can be obtained during the EV charging and/or discharging during the whole day.

#### 4.3.3. Case 1 vs. Case 4

**Figure 7:**
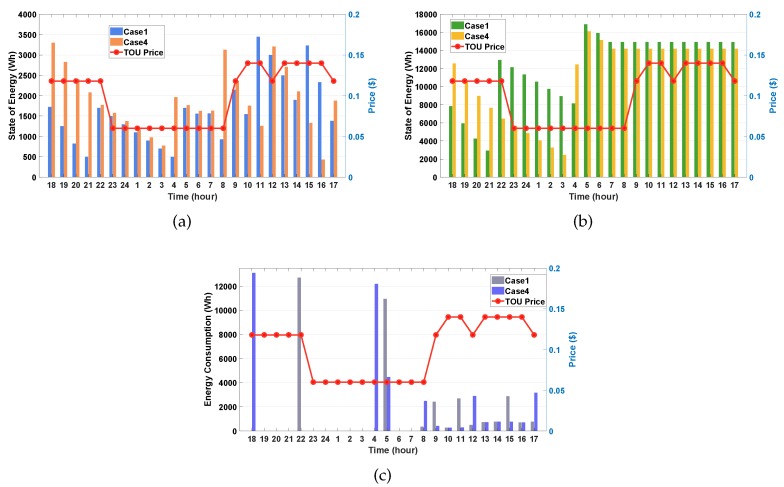
Performance comparison between Case 1 and Case 4. (**a**) SOE of the ESS; (**b**) SOE of the EV; (**c**) net consumption of household.

In this simulation, we investigate and compare the performance between Case 1 (with a high initial SOE) and Case 4 (with a low initial SOE). Figure 7a,b illustrate the SOE schedules of the ESS and EV for Case 1 and Case 4, respectively. First, we observe from Figure 7b that the SOE of the EV at 6:00 p.m. is larger in Case 4 than in Case 1. This is because the low initial SOE of the EV enable the EV to require more charging power to support the upcoming household load demands. This observation is also verified for the ESS as shown Figure 7a where the ESS charges more power in Case 4 than in Case 1 at 6:00 p.m. with the same reason. We also observe from Case 4 in Figure 7c that the highest net energy consumption occurs at 6:00 p.m., owing to a significant charging of the ESS and EV. It is noted that this large amount of the net energy consumption is not observed in previous cases. This is because in these cases the EV has a high initial SOE so that it does not have to charge energy from grid in advance. Except the time slot at 6:00 p.m., the schedules of the net energy consumption at other time slots are similar to the schedules in Case 1.

**Figure 8:**
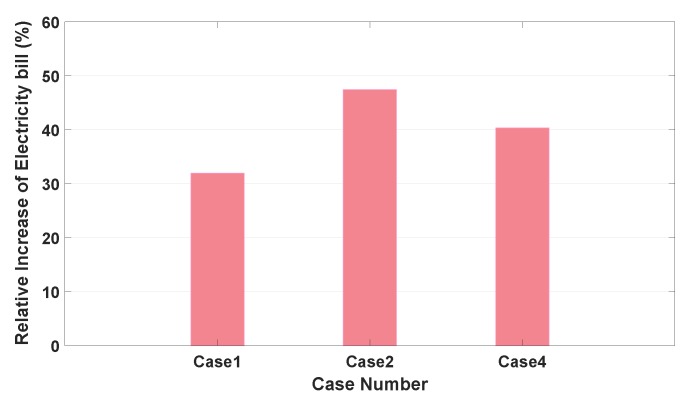
Comparison of a relative increase of the total electricity bill in Case 3 among the considered three cases.

Figure 8 shows a relative increase of the total electricity bill for the aforementioned Cases 1, 2, and 4 with respect to Case 3 with the minimum total electricity bill using the following metric:

(27) $$\frac{X_{n}^{\mathrm{bill}}-X_{3}^{\mathrm{bill}}}{X_{3}^{\mathrm{bill}}}\times 100\left(\%\right),$$

where $X_{3}^{\mathrm{bill}}$ is the total electricity bill for Case 3 and $X_{n}^{\mathrm{bill}}$ is the total electricity bill for Case *n* where n=1, 2, and 4.

Note in this figure that the relative total electricity bills for the three cases are listed in decreasing order of their bills as follows: Case 2 > Case 4 > Case 1. Note in turn from this list that the relative increase of the electricity bill in Case 1 (a sunny weekday with high $SOE_{\mathrm{ini}}^{\mathrm{EV}}$) is smaller than the other two cases (a sunny or rainy weekday with high or low $SOE_{\mathrm{ini}}^{\mathrm{EV}}$). Thus, a fraction of the charging and discharging periods of the EV constitutes the most influential aspect for saving on electricity bills.

**Figure 9:**
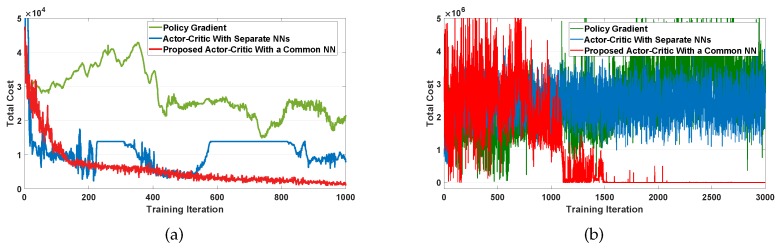
Convergence of the total cost. (**a**) Level 1; (**b**) Level 2.

Figure 9a,b show training curves that present the convergence of the total cost for the first and second levels, respectively. Each figure compares the three training curves using policy gradient method, actor–critic method with separate actor and critic neural networks (NNs), and proposed actor–critic method with a common NN. We observe from Figure 9a,b that policy gradient and actor–critic with separate NNs show a poor performance of the convergence. By contrast, the proposed actor–critic shows that the training curves steadily decrease and then converge to an optimal policy within a moderate training period.

**Figure 10:**
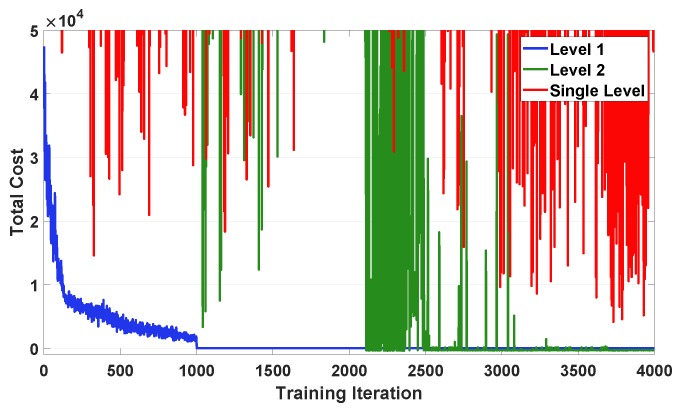
Comparison of the total cost convergence between the proposed two-level DRL approach and the single-level DRL approach.

Figure 10 compares the training curves for the level 1 and level 2 in the proposed DRL method and the single-level DRL method. We can observe from this figure that each training curve for the level 1 and level 2 in the proposed approach steadily decreases and converges to an optimal policy in the training periods of [1, 1,000] and [1,001, 4,000], respectively. By contrast, the training curve for the single-level approach shows a large fluctuation during the training process and a poor value of the result compared to the proposed two-level DRL method. The poor convergence performance of the single-level approach derives from the fact that the complexity of the HEMS problem increases dramatically as the state-action space dimension becomes larger in the single level. Furthermore, since the agent for the EV and ESS is not able to obtain energy consumption data of other appliances in the single level, the single-level approach may not calculate optimal charging and discharging actions of the ESS and EV.

**Figure 11:**
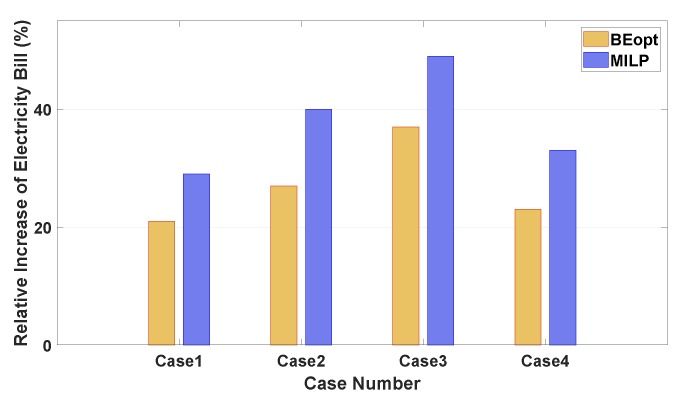
Comparison of a relative increase of the total electricity bill in the proposed DRL method using building energy optimization tool (BEopt) and mixed-integer linear programming (MILP) programs for four cases.

Figure 11 shows a relative increase of the total electricity bill in Cases 1–4 using a Building Energy Optimization Tool (BEopt) [36] and MILP optimization method with respect to the proposed approach. BEopt is widely used as an energy simulation program for the residential building. To fairly compare the performance of the proposed method to that of the MILP method, the MILP method was executed for 24 h with a 1-h scheduling resolution. We can verify from this figure that our proposed method is the most economical for all four cases compared to two existing approaches using BEopt and MILP methods. In particular, it is observed that Case 3 (a sunny weekend with high $SOE_{\mathrm{ini}}^{\mathrm{EV}}$) shows the largest cost increase. This observation implies that the proposed DRL approach schedules the charging and discharging energy of EV in a much more cost-effective way.

The meaningful observations of the proposed HEMS approach in the numerical examples can be summarized as follows:

- Through a comparison between {Case 1, Case 3, Case 4} (with PV system) and Case 2 (without PV system), we conclude that PV generation has a significant impact on the reduction of the total cost of electricity. For example, the total cost of electricity in Case 2 is 11% higher than in Case 1.
- Given that the battery capacity of the EV is approximately four times larger than that of the ESS, the EV can discharge more power than the ESS to cover the total cost of electricity. This can be verified through a comparison between {Case 1, Case 2} (in weekday) and Case 3 on weekends. In contrast, different driving patterns associated with the initial SOE of the EV significantly influence the total cost of electricity. We conclude from a comparison between Case 1 (with high SOE) and Case 4 (with low SOE) that the total cost of electricity in Case 4 is 7% higher than in Case 1. This is because the EV with low SOE needs to charge more power than with high SOE to satisfy the consumer’s preferred SOE at departure time.

## 5. Conclusions

In this study, we propose a two-level distributed deep reinforcement learning algorithm to minimize the cost of electricity through the energy consumption scheduling of two controllable home appliances (an air conditioner and a washing machine) and the charging and discharging of an energy storage system and an electric vehicle while maintaining the consumer’s comfort level and appliance operation characteristics. In the proposed deep reinforcement learning method, two agents interact with each other to schedule the optimal home energy consumption efficiently. One agent for a washing machine and an air conditioner determines their continuous actions in the first level to schedule optimal energy consumption within the consumer’s preferred indoor temperature and operation period, respectively. Based on the optimal energy consumption schedules from the first level, the other agent for an energy storage system and an electric vehicle conducts their continuous charging and discharging actions in the second level to support the aggregated load for controllable and uncontrollable appliances. The comparative case studies under different weather and driving patterns of the electric vehicle with different initial state of energy confirm that the proposed approach can successfully minimize the cost of electricity within the consumer’s preference.

In future work, we plan to develop a multi-agent reinforcement learning algorithm based on a continuous action space that schedules the energy consumption of multiple smart homes with home appliances and distributed energy resources. A key challenge is to design an information exchange scheme between households to minimize the cost of electricity and maintain each consumer’s comfort level. This future work can be implemented using advanced deep reinforcement learning methods such as deep deterministic policy gradient and asynchronous advantage actor–critic methods.
